# Effects of Beetroot Powder with or without L-Arginine on Postprandial Vascular Endothelial Function: Results of a Randomized Controlled Trial with Abdominally Obese Men

**DOI:** 10.3390/nu12113520

**Published:** 2020-11-16

**Authors:** Ellen T. H. C. Smeets, Ronald P. Mensink, Joris Hoeks, Johan de Vogel-Van den Bosch, Robert J. J. Hageman, Peter J. Joris

**Affiliations:** 1 Department of Nutrition and Movement Sciences, NUTRIM School for Nutrition and Translational Research in Metabolism, Maastricht University Medical Center, P.O. Box 616, 6200 Maastricht, The Netherlands; ethc.smeets@maastrichtuniversity.nl (E.T.H.C.S.); r.mensink@maastrichtuniversity.nl (R.P.M.); j.hoeks@maastrichtuniversity.nl (J.H.); 2 Danone Nutricia Research, 3584 Utrecht, The Netherlands; johan.devogel@nutricia.com (J.d.V.-V.d.B.); robert.hageman@nutricia.com (R.J.J.H.)

**Keywords:** beetroot, L-arginine, dietary nitrate, nitric oxide, flow-mediated vasodilation, endothelial function

## Abstract

Background: Through effects on nitric oxide bioavailability, vascular endothelial function is improved after the intake of a high amount of nitrate or L-arginine, but decreased after the intake of a high-fat meal. Therefore, we compared the effects of beetroot powder with or without L-arginine on postprandial brachial artery flow-mediated vasodilation (FMD) after consumption of a high-fat mixed-meal. Methods: Eighteen abdominally obese men completed this randomized, double-blinded, cross-over trial. The study consisted of five test days, each separated by a wash-out period of at least one week. Participants received in random order, a blended meal with a control or nutritional supplement consisting of beetroot powder providing 200 mg nitrate, beetroot with 0.8 g of L-arginine, beetroot with 1.5 g of L-arginine, or 3.0 g of L-arginine. Participants then fasted and 2 h postprandial FMD measurements were performed. Results: No significant differences between meals were observed for postprandial FMD (*p* = 0.45) levels. However, there was a non-significant trend towards a more beneficial postprandial FMD response with the beetroot-containing meals as compared with meals without beetroot. Conclusion: This trial could not provide evidence for beneficial additive effects of a single dose of beetroot powder combined with L-arginine on postprandial endothelial function in abdominally obese men.

## 1. Introduction

Nitric oxide (NO) is a gas that contributes to the maintenance of a healthy endothelial function, as it prevents long-term atherosclerotic disease progression and cardiovascular event rates because it exerts important vasodilatory, anti-inflammatory, antithrombotic, anti-proliferative, and anti-adhesive effects [1,2,3]. A validated non-invasive method to examine cardiovascular health effects of nutritional interventions that are known to affect NO bioavailability is brachial artery flow-mediated vasodilation (FMD) [4,5].

A high-fat meal may lower NO bioavailability, resulting in an impaired postprandial FMD [6]. Since most of the day is spent in a postprandial state, it is important to find strategies to counteract this impaired endothelial function. A possible strategy to increase NO bioavailability is through the ingestion of dietary nitrate that is converted into NO via the endogenous nitrate-nitrite-NO-pathway [7]. Previously, our group has shown that consumption of beetroot juice providing about 500 mg of nitrate improves postprandial FMD two hours after consumption of a high-fat meal challenge in overweight and slightly obese men [8]. Besides nitrate, L-arginine supplementation is another potential strategy to improve NO bioavailability [9]. After ingestion, the semi-essential amino acid L-arginine will be converted into NO via the L-arginine-NO synthase pathway [7]. Specifically, the results of our recent meta-analysis of randomized controlled trials suggest that high-dose L-arginine supplementation improves postprandial FMD in humans [10].

It is important to note that the majority of the trials investigating effects of dietary nitrate or L-arginine on postprandial FMD have been performed with relatively high amounts of these individual components, i.e., 500 to 1488 mg for dietary nitrate and up to 15 g for L-arginine [8,11]. Therefore, it is very difficult to implement these high amounts into dietary patterns, thereby preventing the translation of these beneficial findings into daily practice. However, it is not known if nutritional supplements containing both dietary nitrate and L-arginine also improve postprandial FMD through additive effects on NO bioavailability, which allows supplementation of the individual components at much lower amounts. The present study compared the effects of nutritional supplements providing lower amounts of beetroot with or without L-arginine on postprandial endothelial function, as assessed by FMD, after consumption of a high-fat mixed-meal in apparently healthy abdominally obese men.

## 2. Materials and Methods

### 2.1. Study Population

Twenty-three apparently healthy abdominally obese male volunteers were recruited through advertisements in local newspapers, flyers in the university and public buildings in Maastricht and in the hospital, and among people who had participated in our earlier studies. Important inclusion criteria were: aged between 40 and 70 years, waist circumference ≥ 102.0 cm, stable body weight, no use of anti-hypertensive medication or drugs known to affect lipid or glucose metabolism, and no diabetes, active cardiovascular diseases, or severe medial diseases that might interfere with the study outcomes parameters (e.g., epilepsy, asthma, chronic obstructive pulmonary disease (COPD), inflammatory bowel diseases (IBD), autoinflammatory diseases, or rheumatoid arthritis). Participation in another trial during the past 30 days was also a contraindication. Informed consent was obtained before the start of the study. The protocol was officially approved by the Medical Ethical Committee (METC) of the University Hospital Maastricht (azM) and Maastricht University (UM) and registered at ClinicalTrials.gov in August 2018 as NCT03625596.

### 2.2. Study Design

A randomized, double-blinded, cross-over study was performed. Each participant started with a baseline test day to become familiarized with the measurements, followed by a run-in period of at least four weeks. After the baseline test day, participants were requested to take for the rest of the study daily half a tablet providing vitamins and minerals within the recommended daily allowance (Centrum Original Advanced, PF Consumer Healthcare, The Netherlands) to reduce the risk of nutritional deficiencies. After the run-in period, each participant underwent five postprandial test days. All test days were at least one week apart. To standardize measurements, which were performed at the Metabolic Research Unit Maastricht (MRUM) as much as possible, participants received instructions related to physical activity, food intake, and alcohol consumption as described before [12]. On the morning of the test day, first, an intravenous cannula was placed, followed by an acclimatization period of at least 15 min in a supine position in a quiet and darkened room with a stable temperature of 22 °C. Thereafter, vascular measurements were carried out, and a fasting venous blood sample (T0) was drawn. Next, the participants consumed three times one-third of a liquid high-fat mixed meal. Each portion had to be consumed within 1 min with 2 min breaks. The 2 min break was shortened if a participant was not able to consume one-third within 1 min. Blood samples were taken 15 min (T15), 30 min (T3), 45 min (T45), 60 min (T60), 90 min (T90), 120 min (T120), and 180 min (T180) after consumption. After the T120 blood sample was taken, measurements were repeated.

### 2.3. Meals

Liquid high-fat mixed-meals that provided 3987 kJ (953 kcal) and 55.3 g (52.3 En%) of fat, 93.2 g (39.2 En%) of carbohydrates, and 19.2 g (8.0 En%) of protein were freshly prepared by an independent person on the morning of a test day. A nutritional supplement consisting of (i) 15 g of beetroot powder (Naturex, Avignon, France) providing 200 mg nitrate and 1.2 mg nitrite, (ii) 15 g of beetroot powder with 0.8 g of L-arginine powder (BioKyowa Inc., MO, USA), (iii) 15 g of beetroot powder with 1.5 g of L-arginine powder, or (iv) 3.0 g of L-arginine powder was added. As a control, the participants received the mixed-meal without any nutritional supplement. The composition of the supplements is given in Table S1. Sucrose was added to standardize the macronutrient composition between test meals. Study participants also received one glass of water next to the meal. The total volume of the blended mixed-meals, including the glass of water, was 730 mL. Meals were provided in random order, and both the researcher and participants were blinded to the composition of the supplement. A randomization scheme was made by an independent researcher using www.randomizer.org.

### 2.4. Anthropometric and Vascular Measurements

Waist circumference was assessed during the screening visit. At the start of each test day, a wall-mounted stadiometer was used to assess height, while body weight was determined without shoes and heavy clothing. Waist (i.e., the midline between the superior border of the iliac crest and the inferior margin of the ribs) and hip (i.e., largest part of the hips) circumferences were also measured.

Office brachial systolic blood pressure (SBP), diastolic blood pressure (DBP), and heart rate (HR) were measured using a semi-continuous monitoring device (Omron M7 Intellisense^TM^, Omron, Hoofddorp, The Netherlands). The first measurement was rejected, and the mean of the final three measurements was taken. Assessment of endothelial function was performed using ultrasound echography. For this, a 13–4 MHz linear transducer (MyLab Gamma^TM^, Esaote, Maastricht, The Netherlands) in B-mode was used. All measurements were carried out by the same sonographer (E.S.), as described [12].

### 2.5. Biochemical Analyses

Plasma prepared from blood collected in sodium fluoride (NaF) and Na_2_EDTA (Becton Dickinson, Erembodegem, Belgium) tubes were used for the determination of plasma glucose concentrations (Glucose HK CP, Horiba ABX, Montpellier, France) at all time points. In addition, blood was sampled in serum STT-II advance tubes (Becton Dickinson) for the determination of serum triacylglycerol (TAG; GPO Trinder, Sigma-Aldrich Corp., St. Louis, MO, USA) concentrations with correction for free glycerol at T0, T60, T120, and T180. In addition, serum total (CHOD-PAP method; Roche Diagnostic Systems), high-density lipoprotein cholesterol (HDL-cholesterol; precipitation method (phosphotungstate precipitant); Roche Diagnostics, Mannheim, Germany) and high-sensitivity C-reactive protein (hsCRP; CRP CP, Horiba ABX, Montpellier, France) concentrations at T0 were measured. Fasting serum low-density lipoprotein (LDL)-cholesterol concentrations were calculated using the Friedewald formula [13]. More details regarding the biochemical analyses can be found in our previous study [12].

### 2.6. Statistical Analyses

All data are presented as means and standard deviations (SDs) unless stated otherwise. Before the start of the study, it was calculated that eighteen study participants were needed to detect a true treatment difference of at least 1.75% point in FMD with an intra-subject variability of 2.3% point as observed in our previous trial [8], when an alpha of 0.05, power of 80% and two-sided test were used.

To test for differences in fasting (T0) values between meals, a repeated-measures analysis of variance (ANOVA) was performed. Postprandial FMD, blood pressure, heart rate, and baseline brachial artery diameters were analyzed using linear mixed models with fasting values as a covariate. The effects of the meal, time, and interaction (meal*time) were analyzed using linear mixed models for postprandial plasma glucose and serum TAG concentrations with fasting concentrations as a covariate. If the interaction term was not significant, it was omitted from the model. Post-hoc tests with Bonferroni correction were performed for the pairwise comparison between the meals. The postprandial values of the baseline and control test day were compared using linear mixed models with fasting values as a covariate. To examine the overall effect of adding beetroot powder to a high-fat mixed-meal, a categorical variable (i.e., 0 for meals with beetroot powder and 1 for meals without beetroot powder) was created and added to the model as meal term. A similar categorical variable was created for L-arginine (meals with vs. meals without L-arginine powder). To investigate the effect of the amount of L-arginine, a categorical variable with four values (i.e., 0 for no L-arginine, 1 for 0.8 g L-arginine, 2 for 1.5 g L-arginine and 3 for 3.0 g L-arginine) was created and added to the model as meal term. Moreover, repeated-measures ANOVA was performed to test for differences between incremental areas under the curve (iAUCs) for plasma glucose and serum TAG concentrations. iAUCs were calculated using the trapezoidal rule. Finally, for the control supplement, a paired Student’s t-test was performed to test for differences between fasting (T0) and postprandial (T120) FMD and blood pressure values. The level of significance was set at *p* < 0.05 using two-tailed tests. All analyses were performed using SPSS (V26.0) software for Windows (SPSS Inc., Chicago, IL, USA).

## 3. Results

### 3.1. Study Participants

**Figure 1:**
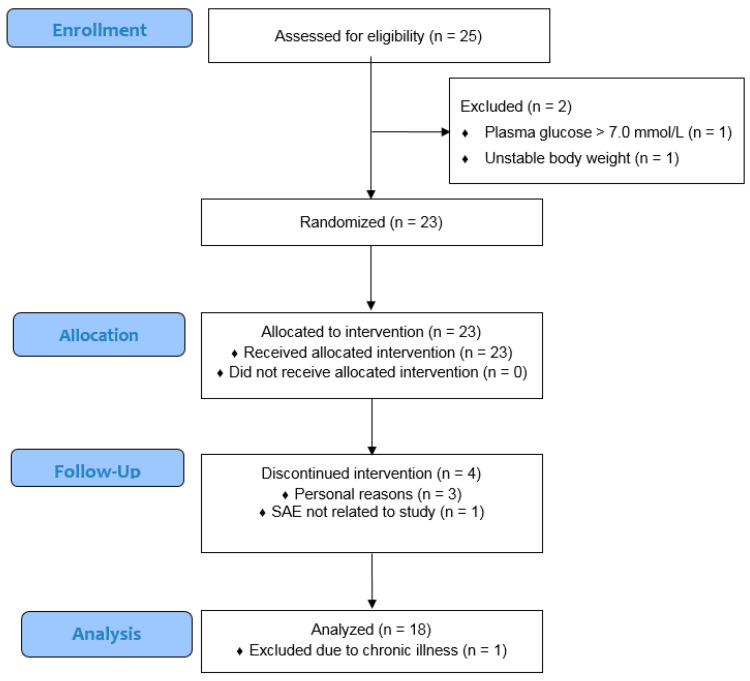
CONSORT flow diagram of the men screened, included, and analyzed in this study.

A total of 25 volunteers were screened, and two men were excluded due to unstable body weight (*n* = 1) or a plasma glucose concentration above 7.0 mmol/L (*n* = 1). Twenty-three participants were randomized, and nineteen participants completed all five test days. Overall, four participants dropped-out because of personal reasons (*n* = 3) or a serious adverse event that was not related to the study (i.e., kidney stones; *n* = 1). One participant was excluded from all statistical analyses due to a chronic disease (i.e., gout) that was not reported during the screening visit but became evident by high plasma hsCRP values during all test days (i.e., 10–57 mg/L). The median age of the eighteen participants that finished the study was 63 (interquartile range (IQR): 61–68.5) years. Their average BMI and waist circumference was 30.8 ± 3.2 kg/m^2^ and 113.0 ± 9.1 cm, respectively. Compliance relating to the daily intake of the half tablet with vitamins and minerals was perfect, with a median intake of 98.7% (IQR: 95.9–100%). One of the participants reported gastrointestinal complaints (i.e., flatulence and diarrhea) following consumption of the meal with beetroot powder without L-arginine, but no other (serious) adverse events were reported during the trial. The CONSORT flow diagram is shown in Figure 1**,** and baseline characteristics are displayed in Table 1.

### 3.2. Vascular and Blood Pressure Measurements 

**Figure 2:**
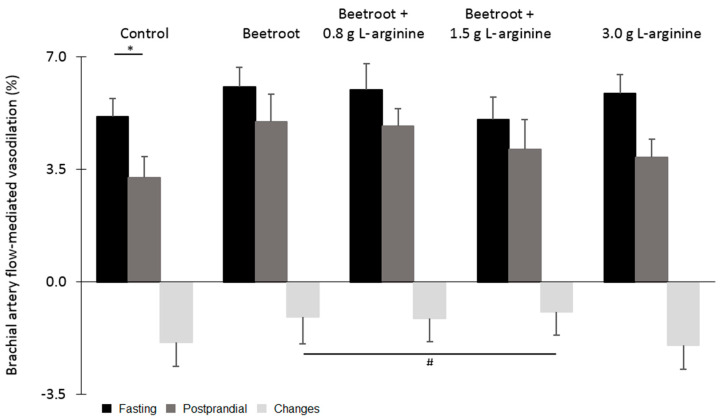
Mean brachial artery flow-mediated vasodilatation (±standard error of the mean (SEM)) before and after the control or nutritional supplements consisting of 15 g beetroot powder, beetroot powder + 0.8 g of L-arginine, beetroot powder + 1.5 g L-arginine, and 3.0 g of L-arginine powder (*n* = 17). * Significantly different from fasting values, *p* < 0.05 (paired Student’s *t*-test). ^#^ Trend difference as compared with other meals, *p* < 0.10 (linear mixed models with fasting values as a covariate). Postprandial FMD values were not significantly affected by the presence (*p* = 0.63) or the amount of L-arginine (*p* = 0.70), respectively (linear mixed models).

No differences were found for the vascular function and blood pressure measures between the baseline and control test day (*p* > 0.05 for all outcomes). FMD could not be analyzed in one participant due to technical difficulties related to the ability of the program to analyze the echo images. Results of the remaining 17 men showed that fasting FMD values (*p* = 0.55, Figure 2) and baseline brachial artery diameters (*p* = 0.59, Table 2) did not differ between meals.

No differences between meals were observed for postprandial FMD and baseline brachial artery diameters (*p* = 0.45 and *p* = 0.99, respectively). There was a non-significant trend towards a more favorable postprandial FMD response after consumption of the beetroot-containing meals as compared with meals without beetroot powder (*p* = 0.074), while postprandial baseline brachial artery diameters did not differ (*p* = 0.91). The presence or the amount of L-arginine did not affect postprandial FMD values (*p* = 0.63 and *p* = 0.70), while postprandial baseline brachial artery diameters were also not affected (*p* = 0.81 and *p* = 0.97, Table 2). Finally, postprandial FMD values were decreased after the meal with the control supplement (*p* = 0.026, Figure 2).

Fasting SBP (*p* = 0.27) and DBP (*p* = 0.44) did not differ between meals. However, fasting HR was significantly different between meals (*p* = 0.017) and tended to be higher before the intake of the control meal as compared with the meal with beetroot and 0.8 g L-arginine powder (*p*= 0.079).

Changes in postprandial brachial SBP, DBP and HR were not different between meals (*p* = 0.82, *p* = 0.92 and *p* = 0.81, respectively). Also, there were no differences in postprandial SBP (*p* = 0.33), DBP (*p* = 0.39) and HR (*p* = 0.65) between meals with and without beetroot or the meals with and without L-arginine powder (*p* = 0.81, *p* = 0.78 and *p* = 0.58, respectively), while the amount of L-arginine did also not affect the results (see Table 2).

### 3.3. Postprandial Lipemia and Glycemia

**Figure 3:**
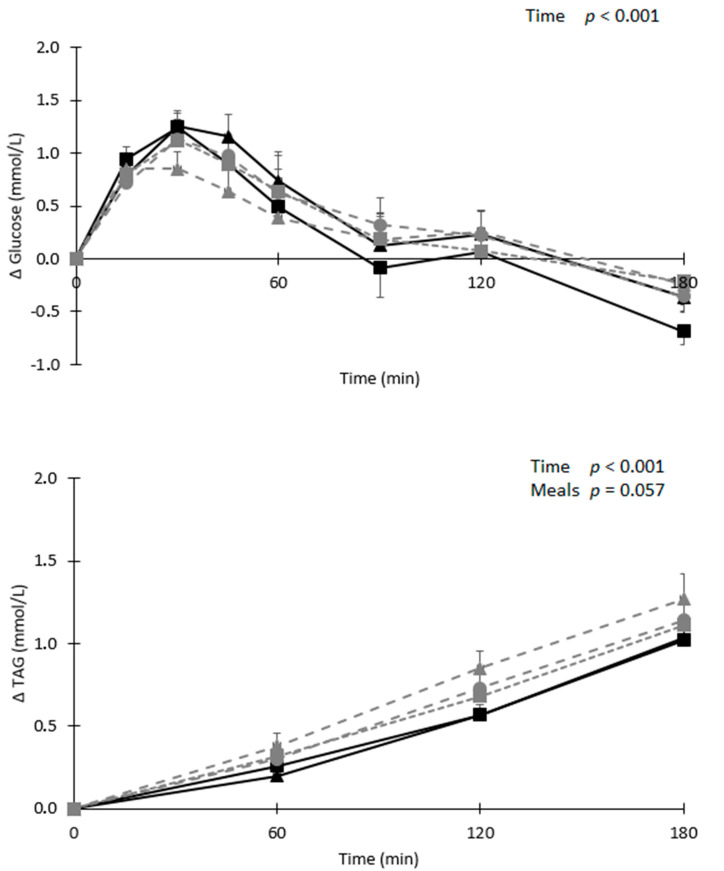
Mean changes (±SEM) in plasma glucose and serum triacylglycerol (TAG) concentrations after consumption of either the control (■), beetroot powder (▲), beetroot powder + 0.8 g L-arginine (●), beetroot powder + 1.5 g L-arginine (■), and 3.0 g L-arginine powder (▲) in a randomized cross-over study in abdominally obese men (*n* = 18). Data were analyzed using linear mixed models with changes from baseline values.

Fasting total cholesterol, HDL-cholesterol, LDL-cholesterol, TAG, glucose, and hsCRP concentrations did not differ between meals (Table 3). After meal consumption, serum TAG concentrations were significantly increased (*p* < 0.001; Figure 3). Furthermore, increases in TAG concentrations tended to differ between meals (*p* = 0.057) with a trend for a higher serum TAG after the consumption of the meal with 3.0 g of L-arginine powder as compared with the meal with beetroot powder (*p* = 0.099). No differences in TAG concentrations were observed between meals with and without beetroot (*p* = 0.19). The presence (*p* = 0.72) or the amount of L-arginine powder (*p* = 0.17) did also not affect changes in serum TAG concentrations. No differences between meals in serum TAG iAUC(_0–180min_) were observed (*p* = 0.10, Table 3).

Postprandial plasma glucose concentrations were significantly increased (*p* < 0.001; Figure 3), but there were no significant differences between meals (*p* = 0.60). In addition, plasma glucose concentrations were not different between meals with and without beetroot or L-arginine powder (*p* = 0.67 and *p* = 0.25, respectively), or when considering different amounts of L-arginine (*p* = 0.26). Plasma glucose iAUC(_0–120min_) and iAUC(_0–180min_) did not differ between meals (*p* = 0.26, and *p* = 0.14, respectively; Table 3).

## 4. Discussion

In this randomized, double-blinded, cross-over study with healthy, abdominally obese men, nutritional supplements containing beetroot and/or various amounts of L-arginine powder did not differently affect postprandial FMD when consumed with a high-fat mixed-meal. However, there was a non-significant trend towards a more beneficial postprandial FMD response with beetroot-containing meals as compared with the meals without beetroot powder. Observed effects on FMD could not be explained by differences in effects on postprandial baseline brachial artery diameters, blood pressure, lipemia, or glycemia.

Using a similar experimental design, our group has earlier shown that beetroot juice, providing about 500 mg of nitrate, improved postprandial FMD values by 1.6 percentage points [8]. In the present study, a non-significant trend towards an improved postprandial FMD by 1.1 percentage points was found after consumption of the beetroot-containing meals as compared with the meals without beetroot powder. This difference in effect may be explained by the 2.5 times lower amount of nitrate provided by the beetroot powder in the present study (200 mg, which can be provided by ~120 g of cooked beetroot, 50 g of rocket salad, or 90 g of uncooked spinach [14,15]) as compared with our previous study [8]. It was noteworthy that the effects of the control supplements on postprandial FMD were comparable between both studies and therefore did not confound the comparison. Other studies observing beneficial effects on postprandial FMD following the intake of beetroot or nitrate salts even provided higher amounts of nitrate (i.e., 500 to 1488 mg) [11,16]. These amounts even exceeded the acceptable daily intake for nitrate of 3.7 mg per kilogram body weight per day [17], which is about 300 mg for an 80 kg adult. Thus these higher amounts may not be suitable for longer-term supplementation.

Beetroot also contains a substantial amount of potassium (~365 mg per 120 g cooked beetroot). Potassium is known to exert vasodilatory properties that may affect vascular function [18]. However, a meta-analysis observed that high-dose potassium supplementation did not affect FMD [18]. As even higher doses of potassium did not affect FMD, we assume that the relatively low amount of potassium present in our beetroot powder could not have influenced our results.

Another important difference between this study and previous studies [8,11,16,19,20] is that high-fat meals were supplemented with beetroot powder instead of beetroot juice or potassium nitrate. To our knowledge, no studies have compared side-by-side the effect on FMD of beetroot powder with those of other nitrate-rich sources. However, there are no obvious reasons that the source of nitrate affects its bioavailability. In fact, consumption of a high-fat meal supplemented with beetroot juice or potassium nitrate powder, in equivalent dosages of nitrate, increased plasma and saliva NOx concentrations to the same extent in older overweight and obese adults [21]. Although effects on FMD have not been investigated so far, differential effects on postprandial FMD between beetroot powder and other sources of nitrate are not expected.

Like nitrate, L-arginine supplementation may also increase NO bioavailability. Previous studies have reported that L-arginine intake improved postprandial FMD after 1.5 to 2 h when added to a high-fat meal [22,23,24], as also shown in our recent meta-analysis [10]. The amount of L-arginine in the studies included ranged from 2.5 to 15 g, with an average intake of 7.8 g of L-arginine, which can be provided by about 130 g of protein [25]. Thus, the amount provided in these studies were substantially higher as compared with the current study. One exception is the study of Borucki and colleagues [22], which used 2.5 g of L-arginine, which is comparable to the highest amount of L-arginine in this study, as part of a high-fat mixed-meal and found an improvement in 2 h postprandial FMD. However, Borucki et al. [22] studied healthy adults who have a higher endothelial nitric oxide synthase (eNOS) protein content and thus an increased capacity for NO production as compared with abdominally obese volunteers [26]. Thus, the beneficial effects of L-arginine may be more pronounced in healthy participants.

Finally, no significant differences between test meals were found for postprandial baseline brachial artery diameters, serum TAG, and plasma glucose concentrations or brachial SBP, DBP, and HR. Thus, these parameters could not have affected our findings.

One limitation of this study is that only men participated; to exclude any possible sex effects, thereby reducing the external validity. A strength is that the effect of different nutritional supplements was examined in a randomized, double-blinded, cross-over design. Moreover, the amount of nitrate supplemented was substantially lower compared with previous studies and within the acceptable daily intake. Future studies should, therefore, examine the longer-term additive effects of lower amounts of nitrate and L-arginine in both men and women.

In conclusion, this randomized-controlled, double-blinded, cross-over study in abdominally obese men provided no evidence for beneficial additive effects of a single dose of beetroot powder combined with various amounts of L-arginine on postprandial endothelial function following the consumption of a high-fat mixed-meal. This lack of effect could not be explained by different changes in postprandial brachial artery diameters, blood pressure, lipemia, or glycemia. However, a trend was found for an improved postprandial endothelial function after consumption of the beetroot-containing meals as compared with the mixed-meals without beetroot powder.

## Figures and Tables

**Table 1:** Baseline characteristics of the eighteen abdominally obese men who completed the study ^a^.

Variables	Study Participants
Age (years)	63 (61–68.5) ^b^
BMI (kg/m^2^)	30.8 ± 3.2
Waist circumference (cm)	113.0 ± 9.1
Hip circumference (cm)	110.5 ± 4.8
Fasting serum total cholesterol (mmol/L)	5.31 ± 0.95
Fasting plasma glucose (mmol/L)	5.88 ± 0.55

^a^ Values are means ± standard deviation (SD). ^b^ Value is the median ± interquartile range (IQR).

**Table 2:** Changes in baseline brachial artery diameters, SBP, DBP, and HR of the abdominally obese men who completed the study ^a,b^.

Variables		Control	Beetroot	Beetroot + 0.8 g L-Arginine	Beetroot + 1.5 g L-Arginine	3.0 g L-Arginine
Baseline brachial artery diameter, mm	Fasting	5.1 ± 0.4	5.0 ± 0.5	5.0 ± 0.5	5.0 ± 0.5	5.0 ± 0.5
	Postprandial	5.1 ± 0.4	5.1 ± 0.5	5.1 ± 0.5	5.1 ± 0.5	5.1 ± 0.5
	Changes	0.0 ± 0.2	0.1 ± 0.2	0.1 ± 0.1	0.1 ± 0.2	0.0 ± 0.1
SBP, mmHg	Fasting	135.3 ± 10.0	136.9 ± 11.6	134.8 ± 11.6	134.4 ± 11.2	136.4 ± 12.9
	Postprandial	134.2 ± 9.7	132.8 ± 9.7	132.5 ± 10.6	132.3 ± 10.9	134.3 ± 11.6
	Changes	−1.2 ± 7.6	−4.1 ± 7.0	–2.3 ± 5.7	−2.1 ± 9.2	−2.1 ± 7.7
DBP, mmHg	Fasting	82.6 ± 6.4	83.1 ± 6.3	81.8 ± 7.6	82.2 ± 6.7	83.5 ± 6.7
	Postprandial	80.1 ± 7.4	79.6 ± 6.9	78.2 ± 7.6	79.1 ± 7.6	80.3 ± 7.9
	Changes	−2.5 ± 5.2	−3.5 ± 5.7	−3.7 ± 5.2	−3.1 ± 5.9	−3.2 ± 5.3
HR, beats/min	Fasting	63.4 ± 9.7	61.9 ± 8.5	60.6 ± 7.9	60.6 ± 9.3	61.1 ± 11.6
	Postprandial	61.8 ± 9.2	60.8 ± 8.0	59.4 ± 7.2	60.6 ± 8.6	60.4 ± 9.8
	Changes	−1.6 ± 5.1	−1.1 ± 2.5	−1.1 ± 3.3	−0.1 ± 3.3	−0.7 ± 4.4

^a^ Values are means ± SD. ^b^ DBP: diastolic blood pressure; HR: heart rate; SBP: systolic blood pressure.

**Table 3:** Fasting total cholesterol, HDL-cholesterol, LDL-cholesterol, TAG, glucose and hsCRP concentrations and postprandial iAUC values for glucose and TAG of the eighteen abdominally obese men who completed the study ^a,b^.

Variables	Control	Beetroot	Beetroot + 0.8 g L-Arginine	Beetroot + 1.5 g L-Arginine	3.0 g L-Arginine
Total cholesterol (mmol/L)	5.03 ± 1.13	5.02 ± 1.20	4.97 ± 1.13	5.07 ± 1.16	4.98 ± 1.14
HDL-cholesterol (mmol/L)	1.15 ± 0.23	1.13 ± 0.24	1.16 ± 0.22	1.15 ± 0.25	1.16 ± 0.22
LDL-cholesterol (mmol/L)	3.55 ± 1.01	3.56 ± 1.05	3.53 ± 1.04	3.61 ± 1.07	3.53 ± 1.07
TAG (mmol/L)	1.68 ± 1.54	1.64 ± 1.49	1.42 ± 0.98	1.53 ± 1.21	1.48 ± 1.20
Glucose (mmol/L)	6.01 ± 0.42	6.03 ± 0.49	5.95 ± 0.37	5.99 ± 0.40	5.98 ± 0.44
hsCRP (mg/L) ^c^	1.44 (0.63–2.68)	1.29 (0.58–2.59)	0.93 (0.67–2.82)	1.39 (0.94–2.93)	1.54 (0.81–3.14)
iAUC glucose (mmol/L/120 min)	130.6 ± 49.9	125.7 ± 47.7	114.3 ± 50.9	122.7 ± 48.4	108.3 ± 50.6
iAUC glucose (mmol/L/180 min)	213.0 ± 112.3	183.6 ± 65.1	170.4 ± 67.0	161.2 ± 59.3	176.0 ± 100.4
iAUC TAG (mmol/L/180 min)	80.4 ± 35.9	79.9 ± 37.9	105.9 ± 85.3	93.1 ± 42.4	112.1 ± 58.8

^a^ Values are means ± SD. ^b^ iAUC: incremental area under the curve; HDL: high-density lipoprotein; hsCRP: high-sensitivity C-reactive protein; LDL: low-density lipoprotein; TAG: triacylglycerol. ^c^ Values are median ± IQR.

## Supplementary Materials

The following are available online at https://www.mdpi.com/2072-6643/12/11/3520/s1, Table S1: The amount of L-arginine, nitrate, nitrite and sucrose added to the high-fat mixed-meals.
