# Supplementary material for: Effects of Beetroot Powder with or without L-Arginine on Postprandial Vascular Endothelial Function: Results of a Randomized Controlled Trial with Abdominally Obese Men
Source: Nutrients. 2020 Nov 16;12(11):3520. doi: 10.3390/nu12113520 (PMC7697292; doi:10.3390/nu12113520)
Supplement: Supplementary file 1 [file nutrients-12-03520-s001.zip › Nutrients_Tables_E_Smeets.pdf]

Table 1 Baseline characteristics of the eighteen abdominally obese men who completed the study[a].

**Variables**	**Study participants**
Age (years)	63 (61 – 68.5)[b]
BMI (kg/m^2)	30.8 ± 3.2
Waist circumference (cm)	113.0 ± 9.1
Hip circumference (cm)	110.5 ± 4.8
Fasting serum total cholesterol (mmol/L)	5.31 ± 0.95
Fasting plasma glucose (mmol/L)	5.88 ± 0.55

[a] Values are means ± SD.

[b] Value is the median ± IQR.

Table 2 Changes in baseline brachial artery diameters, SBP, DBP and HR of the abdominally obese men who completed the study[a,b].

Variables		Control	Beetroot	Beetroot + 0.8 g L-arginine	Beetroot + 1.5 g L-arginine	3.0 g L-arginine
Baseline brachial artery diameter, mm	Fasting	5.1 ± 0.4	5.0 ± 0.5	5.0 ± 0.5	5.0 ± 0.5	5.0 ± 0.5
	Postprandial	5.1 ± 0.4	5.1 ± 0.5	5.1 ± 0.5	5.1 ± 0.5	5.1 ± 0.5
	Changes	0.0 ± 0.2	0.1 ± 0.2	0.1 ± 0.1	0.1 ± 0.2	0.0 ± 0.1
SBP, mmHg	Fasting	135.3 ± 10.0	136.9 ± 11.6	134.8 ± 11.6	134.4 ± 11.2	136.4 ± 12.9
	Postprandial	134.2 ± 9.7	132.8 ± 9.7	132.5 ± 10.6	132.3 ± 10.9	134.3 ± 11.6
	Changes	-1.2 ± 7.6	-4.1 ± 7.0	-2.3 ± 5.7	-2.1 ± 9.2	-2.1 ± 7.7
DBP, mmHg	Fasting	82.6 ± 6.4	83.1 ± 6.3	81.8 ± 7.6	82.2 ± 6.7	83.5 ± 6.7
	Postprandial	80.1 ± 7.4	79.6 ± 6.9	78.2 ± 7.6	79.1 ± 7.6	80.3 ± 7.9
	Changes	-2.5 ± 5.2	-3.5 ± 5.7	-3.7 ± 5.2	-3.1 ± 5.9	-3.2 ± 5.3
HR, beats/min	Fasting	63.4 ± 9.7	61.9 ± 8.5	60.6 ± 7.9	60.6 ± 9.3	61.1 ± 11.6
	Postprandial	61.8 ± 9.2	60.8 ± 8.0	59.4 ± 7.2	60.6 ± 8.6	60.4 ± 9.8
	Changes	-1.6 ± 5.1	-1.1 ± 2.5	-1.1 ± 3.3	-0.1 ± 3.3	-0.7 ± 4.4

[a] Values are means ± SD

[b] DBP: diastolic blood pressure; HR: heart rate; SBP: systolic blood pressure.

Table 3 Fasting total cholesterol, HDL-cholesterol, LDL-cholesterol, TAG, glucose and hsCRP concentrations and postprandial iAUC values for glucose and TAG of the eighteen abdominally obese men who completed the study[a,b].

Variables	Control	Beetroot	Beetroot + 0.8 g L-arginine	Beetroot + 1.5 g L-arginine	3.0 g L-arginine
Total cholesterol (mmol/L)	5.03 ± 1.13	5.02 ± 1.20	4.97 ± 1.13	5.07 ± 1.16	4.98 ± 1.14
HDL-cholesterol (mmol/L)	1.15 ± 0.23	1.13 ± 0.24	1.16 ± 0.22	1.15 ± 0.25	1.16 ± 0.22
LDL-cholesterol (mmol/L)	3.55 ± 1.01	3.56 ± 1.05	3.53 ± 1.04	3.61 ± 1.07	3.53 ± 1.07
TAG (mmol/L)	1.68 ± 1.54	1.64 ± 1.49	1.42 ± 0.98	1.53 ± 1.21	1.48 ± 1.20
Glucose (mmol/L)	6.01 ± 0.42	6.03 ± 0.49	5.95 ± 0.37	5.99 ± 0.40	5.98 ± 0.44
hsCRP (mg/L)[c]	1.44 (0.63 – 2.68)	1.29 (0.58 – 2.59)	0.93 (0.67 – 2.82)	1.39 (0.94 – 2.93)	1.54 (0.81 – 3.14)
iAUC glucose (mmol/L/120min)	130.6 ± 49.9	125.7 ± 47.7	114.3 ± 50.9	122.7 ± 48.4	108.3 ± 50.6
iAUC glucose (mmol/L/180min)	213.0 ± 112.3	183.6 ± 65.1	170.4 ± 67.0	161.2 ± 59.3	176.0 ± 100.4
iAUC TAG (mmol/L/180min)	80.4 ± 35.9	79.9 ± 37.9	105.9 ± 85.3	93.1 ± 42.4	112.1 ± 58.8

[a] Values are means ± SD

[b] iAUC: incremental area under the curve; HDL: high-density lipoprotein; hsCRP: high-sensitivity C-reative protein; LDL: low-density lipoprotein; TAG: triacylglycerol.

[c] Values are median ± IQR
