# Supplementary figures and images for: Effects of Beetroot Powder with or without L-Arginine on Postprandial Vascular Endothelial Function: Results of a Randomized Controlled Trial with Abdominally Obese Men
Source: Nutrients. 2020 Nov 16;12(11):3520. doi: 10.3390/nu12113520 (PMC7697292; doi:10.3390/nu12113520)

Enrollment

Allocation

Follow-Up

Analysis

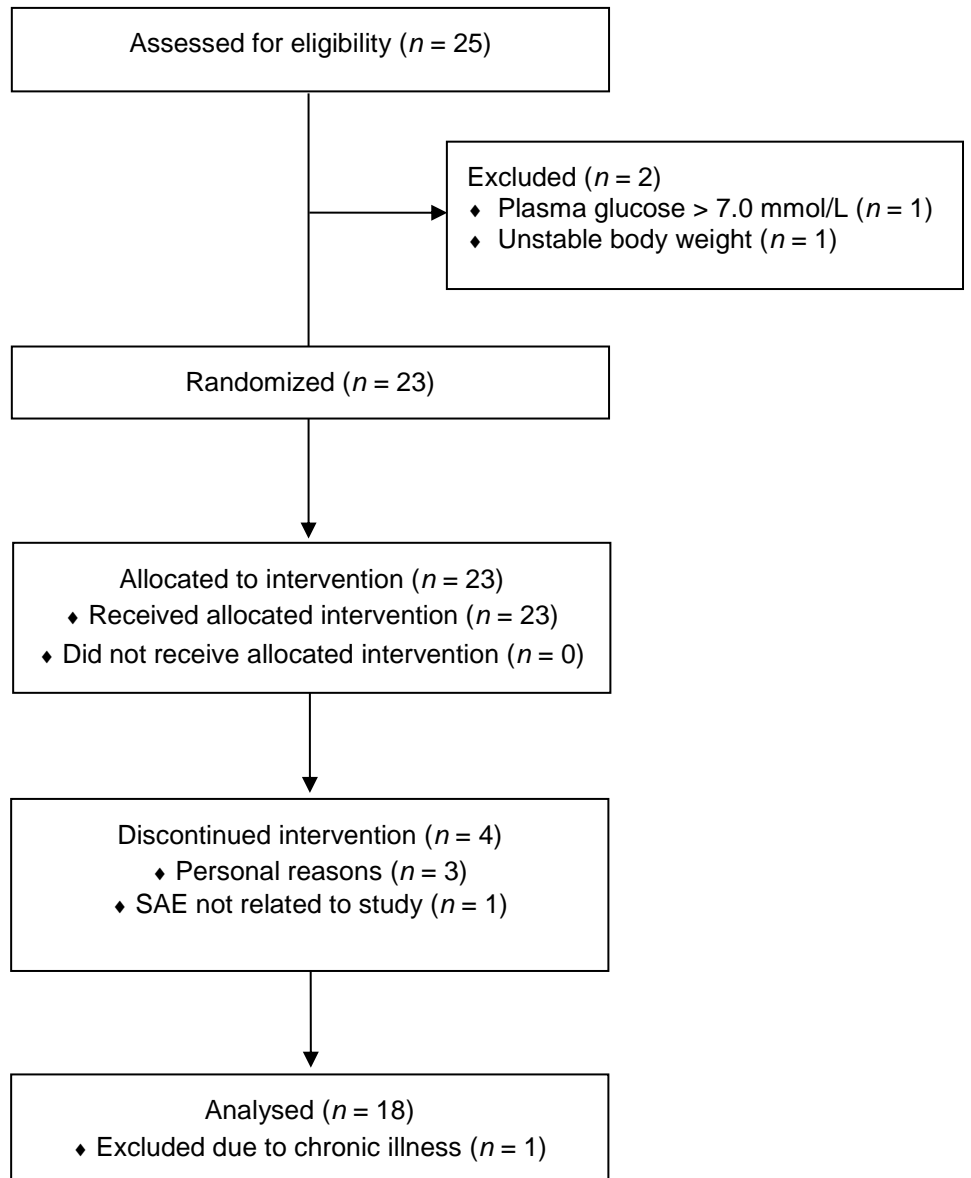

Fig1

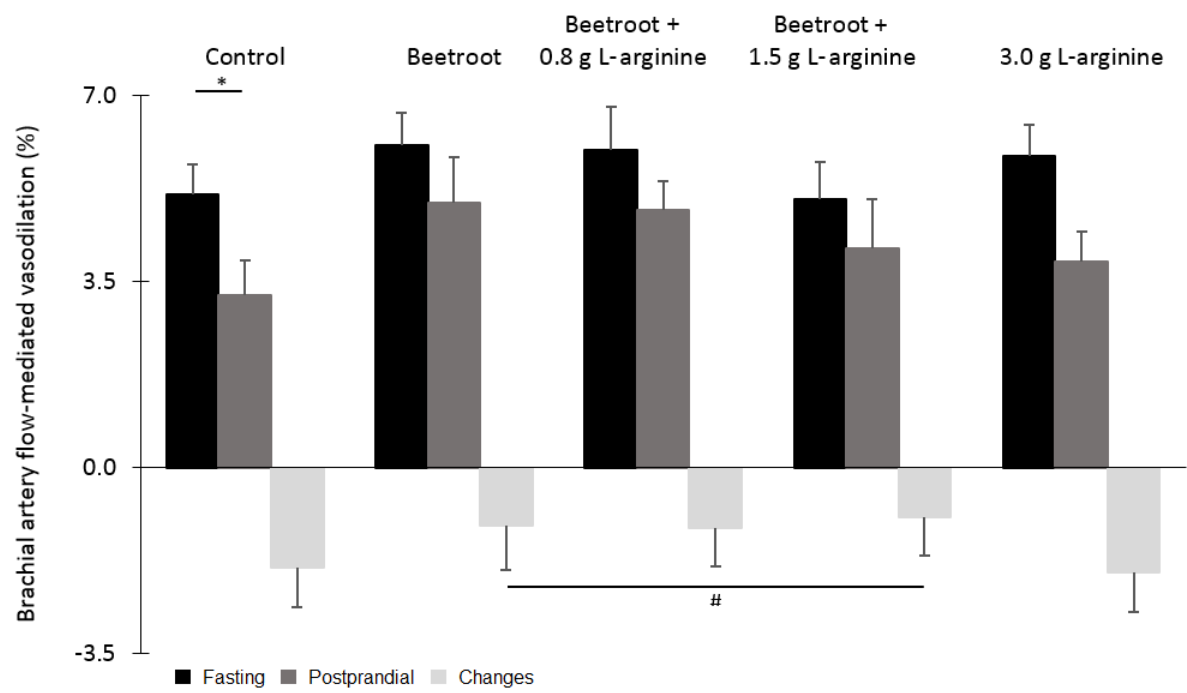

**Fig 2**

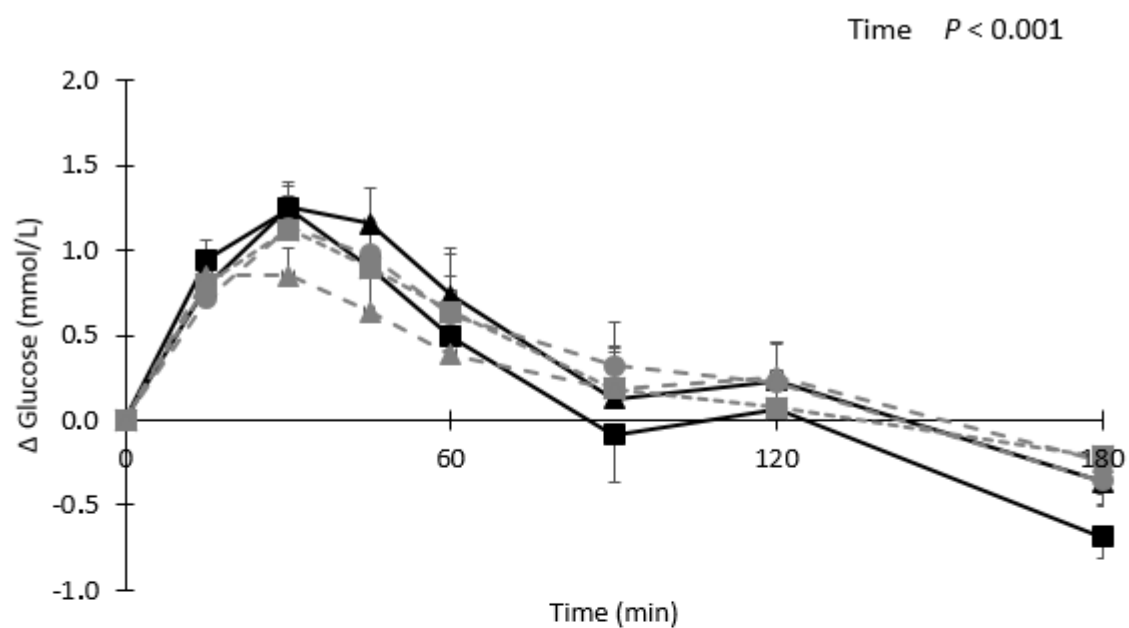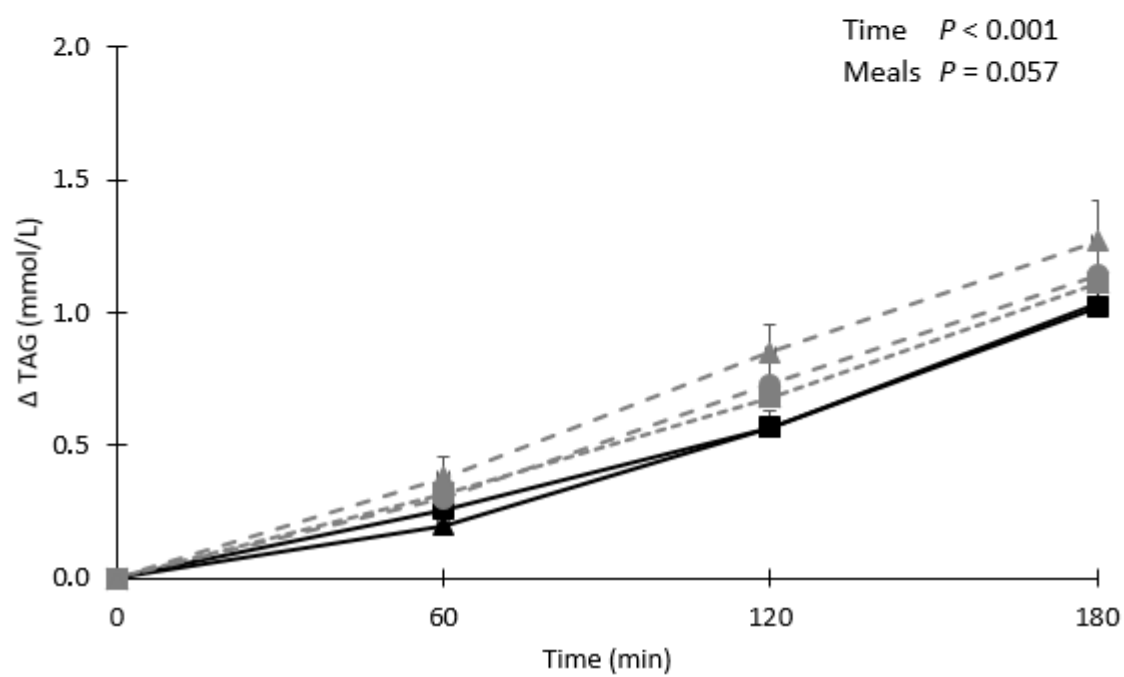

30

31 **Fig 3**

Supplement: Supplementary file 1 [file nutrients-12-03520-s001.zip › Nutrients_Figures_E_Smeets.pdf]
